# Supplementary material for: Violence, runaway, and suicide attempts among people living with schizophrenia in China: Prevalence and correlates
Source: PeerJ. 2022 Mar 1;10:e13033. doi: 10.7717/peerj.13033 (PMC8896021; doi:10.7717/peerj.13033)
Supplement: Supplemental Information 2 [file peerj-10-13033-s002.docx]

**Table 1 risk behaviors

replace drugadh=2 if drugadh==1

replace drugadh=1 if drugadh==0

replace drugadh=0 if drugadh==2

label values drugadh yn

tab drugadh

replace inhospital=0 if inhospital==1 |inhospital==.

replace inhospital=1 if inhospital==2

label define risklbl 0"no" 1"yes"

label values inhospital risklbl

tab inhospital, missing

replace suicide=0 if suicide ==1 |suicide==.

replace suicide=1 if suicide==2

label values suicide risklbl

tab suicide, missing

replace damage=0 if damage ==1 |damage==.

replace damage=1 if damage==2

label values damage risklbl

tab damage, missing

replace beat=0 if beat ==1 |beat==.

replace beat=1 if beat==2

label values beat risklbl

tab beat, missing

replace runaway=0 if runaway ==1 |runaway==.

replace runaway=1 if runaway==2

label values runaway risklbl

tab runaway, missing

gen violence=1 if damage==1|beat==1

replace violence=0 if violence==.

label values violence risklbl

tab violence, missing

gen risk=1 if suicide==1|damage==1|beat==1|runaway==1

replace risk=0 if risk==.

label values risk risklbl

tab risk, missing

gen risk1=1 if suicide==1|damage==1|beat==1|runaway==1

replace risk1=0 if risk1==.

gen nrisk= suicide+ damage+ beat+ runaway

sum nrisk, d

tab nrisk

gen nrisk1=violence+suicide+runaway

replace nrisk1=. if nrisk1==0

tab nrisk1 sex , chi column

tab1 inhospital suicide damage beat runaway risk nrisk

**no gender difference in violent behaviors

tab risk sex , chi column

tab violence sex , chi column

tab suicide sex , chi column

tab damage sex , chi column

tab beat sex , chi column

tab runaway sex , chi column

tab nrisk sex , chi column

***************

***Table 2 univariate analysis

**************

sum age BST BS1 BS2 BS3 BS4 BS5 tphq tgad tdiasbility GAF

tab1 sex marriage1 education1 work1 drugadh inhospital phqcat2 gadcat2

***socio-demographics and risk behaviors

ttest age, by(risk)

tab sex risk, chi column

tab marriage1 risk, chi column

tab education1 risk, chi column

tab work1 risk, chi column

***clinical and risk behaviors

tab drugadh risk, chi column

tab inhospital risk, chi column

tab phqcat2 risk, chi column

tab gadcat2 risk, chi column

ttest BST, by(risk)

ttest BS1, by(risk)

ttest BS2, by(risk)

ttest BS3, by(risk)

ttest BS4, by(risk)

ttest BS5, by(risk)

ttest tphq, by(risk)

ttest tgad, by(risk)

ttest tdiasbility, by(risk)

ttest GAF, by(risk)

***multivariate analysis

logistic risk age i.sex i.marriage1 i.education1 i.work1 i.drugadh i. inhospital BST i.phqcat2 i.gadcat2 tdiasbility GAF
