# Supplementary material for: Violence, runaway, and suicide attempts among people living with schizophrenia in China: Prevalence and correlates
Source: PeerJ. 2022 Mar 1;10:e13033. doi: 10.7717/peerj.13033 (PMC8896021; doi:10.7717/peerj.13033)
Supplement: Supplemental Information 3 [file peerj-10-13033-s003.docx]

**Table1. Full name or explanation of the variable names of the original dataset**

| **Variable name** | **Full name/explanation** |
| --- | --- |
| age | age of participants |
| sex | gender of participants |
| marriage1 | marital status of participants |
| education1 | education level of participants |
| work1 | employment status of participants |
| inhospital | whether have involuntary hospital admission |
| drugadh | medication adherence |
| tdiasbility | the total score of the 12-item World Health Organization Disability Assessment Schedule 2.0 (WHODAS 2.0) |
| tphq | the total score of the 9-item Patient Health Questionnaire-9 (PHQ-9) |
| phqcat2 | whether have depression based on a cutoff of 10 for the PHQ-9 |
| tgad | the total score of the 7-item Generalized Anxiety Disorder Scale-7 (GAD-7) |
| gadcat2 | whether have depression based on a cutoff of 10 for the GAD-7 |
| BST | the total score of the 18-item Brief Psychiatric Rating Scale (BPRS-18) |
| BS1 | the total score of the BPRS-18 subscale: affect |
| BS2 | the total score of the BPRS-18 subscale: positive symptoms |
| BS3 | the total score of the BPRS-18 subscale: negative symptoms |
| BS4 | the total score of the BPRS-18 subscale: resistance |
| BS5 | the total score of the BPRS-18 subscale: activation |
| GAF | the total score of the Global Assessment of Functioning (GAF) |
